# Effects of Wind–Water Erosion and Topographic Factor on Soil Properties in the Loess Hilly Region of China

**DOI:** 10.3390/plants12132568

**Published:** 2023-07-06

**Authors:** Dengfeng Tuo, Qi Lu, Bo Wu, Qiang Li, Bin Yao, Leilei Cheng, Jinlei Zhu

**Affiliations:** 1 Institute of Ecological Conservation and Restoration, Chinese Academy of Forestry, Beijing 100091, China; 2 Institute of Desertification Studies, Chinese Academy of Forestry, Beijing 100091, China; 3 Shaanxi Key Laboratory of Ecological Restoration in Shaanbei Mining Area, Yulin University, Yulin 719000, China

**Keywords:** wind erosion, water erosion, topographic factor, ^137^Cs inventory, soil properties

## Abstract

Wind and water erosion processes can lead to soil degradation. Topographic factors also affect the variation of soil properties. The effect of topographic factors on soil properties in regions where wind and water erosion simultaneously occur remains complicated. To address this effect, we conducted this study to determine the relationships between the changes in wind–water erosion and soil properties in different topographic contexts. We collected soil samples from conical landforms with different slope characteristics and positions in the wind–water erosion crisscross region of China. We examined the soil ^137^Cs inventory, soil organic carbon (SOC), total nitrogen (TN), soil particles, soil water content (SWC), and biomass. ^137^Cs was applied to estimate soil erosion. The results show that the soil erosion rate followed the order of northwest slope > southwest slope > northeast slope > southeast slope. The soil erosion rate on the northwest slope was about 12.06–58.47% higher than on the other. Along the slopes, the soil erosion rate decreased from the upper to the lower regions, and was 65.65% higher at the upper slope than at the lower one. The change in soil erosion rate was closely related to soil properties. The contents of SOC, TN, clay, silt, SWC, and biomass on the northern slopes (northwest and northeast slopes) were lower than those on the southern slopes (southeast and southwest slopes), and they were lower at the upper slope than at the lower one. Redundancy analysis showed that the variation in soil properties was primarily affected by the slope aspect, and less affected by soil erosion, accounting for 56.1% and 30.9%, respectively. The results demonstrate that wind–water erosion accelerates the impact of topographic factors on soil properties under slope conditions. Our research improves our understanding of the mechanisms of soil degradation in gully regions where wind and water erosion simultaneously occur.

## 1. Introduction

Soil erosion is a widespread environmental problem and can lead to significant soil degradation in sloping fields [1,2,3]. Erosion involves the dynamic processes of fine soil particles being denuded and transported and soil nutrients adhering to the particles, which are consequentially lost together [4,5,6]. Wind and water erosion are the two dominant erosion types and substantially interact in arid and semiarid regions [7,8]. The amount of soil lost through erosion via wind and water is known to be higher than that lost as a result of either single erosion type [9]. Understanding the effect of wind and water erosion on soil properties is crucial for unravelling the soil degradation process in arid and semiarid regions [10].

A scientific understanding of the variation in soil properties in wind and water erosion environments in hilly and gully landforms requires a consideration of topographical factors. Wind erosion is determined mainly by wind direction, while water erosion is affected by overland flow and raindrop impact [11,12]; as such, wind and water erosion differ between environments with different slope aspects and positions [13]. Beullens et al. [11] selected six barren conical spoil heaps in Belgium and northern France, and found that the dominant wind in this area comes from the southwestward direction, which brings a large amount of rain with it and leads to the southwest slope suffering the highest rill erosion rates. On the Chinese Loess Plateau, Zhang et al. [13] reported that the dominant wind direction was mainly northwestward, while the southeast slope experienced almost no wind erosion, but suffered pronounced water erosion.

Topography also directly affects the variation in soil properties through biotic and abiotic processes [14,15,16]. When there are different slope aspects and positions, the ground surface receives unequal amounts of solar radiation and soil evaporation, with a significant effect on soil moisture, thus further affecting microbial activity [17]. Higher moisture conditions could increase plant residues. As a result, the chemical compositions of plant residues and microbial transformations are the major prerequisites of soil carbon and nitrogen formation [18]. To date, a wide range of studies have reported the effects of topographic factors or soil erosion caused by wind or water on soil properties, but few research efforts have clarified the relationships between wind and water erosion, topographic factors, and soil properties. The effects of topographic factors in relation to wind–water erosion on soil properties remain elusive, limiting our understanding of soil degradation mechanisms. 

Caesium-137 (^137^Cs) is an artificial radionuclide (half-life of 30.17 years) that was released into the environment after the nuclear weapons tests undertaken primarily during the 1950–1970s. ^137^Cs has been widely applied as a surrogate in soil erosion studies on almost every continent [19,20]. The use of ^137^Cs to estimate erosion and deposition rates is based on the comparison of this radiotracer in sampling sites with the data in the reference inventory on the local input of ^137^Cs—the inventory in study sites should correspond to sites affected by erosion and/or sediment deposition, and the reference should correspond to that of a site that has not been affected over the last 53 year [21,22,23].

In this study, we focused on the conical landforms in the wind–water erosion crisscross region of the Chinese Loess Plateau. These are higher than the surrounding landforms; in this context, all aspects of the slope are exposed, meaning the weather conditions have a direct influence on them, and thus, soils at the surface are subject to erosion by wind and water. ^137^Cs was used to estimate soil erosion. The objectives of this study were to: (1) quantify the wind and water erosion rates in different slope aspects and positions; (2) investigate the topographic factor, as well as wind and water erosion, and their interaction effects on soil properties; (3) quantify the contributions of wind–water erosion and the topographic factor to the variation in soil properties. The results will improve our understanding of soil degradation mechanisms and help researchers develop strategies to reduce soil erosion and nutrient losses in a hilly region of the Loess Plateau in China. 

## 2. Results

### 2.1. Effects of Topographic Factor on Soil ^137^Cs Inventory and Erosion Rate

**Figure 1:**
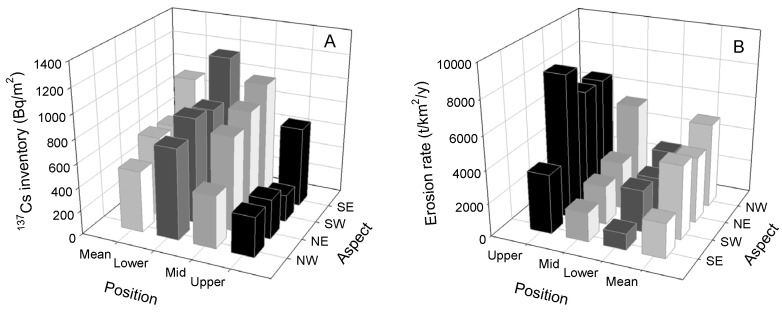
Soil (**A**) ^137^Cs inventory and (**B**) erosion rate under different topographic conditions. NW: northwest; NE: northeast; SW: southwest; SE: southeast.

A descriptive statistical summary of the soil ^137^Cs inventory is shown in Table 1. The soil ^137^Cs inventory varied greatly. The slope aspect was the factor that most significantly reflected the effects on the soil ^137^Cs inventory and soil erosion (Table 2). As shown in Figure 1A, the highest ^137^Cs level was identified on the southeast slope (947 Bq/m^2^), followed by the northeast slope (671 Bq/m^2^), the southwest slope (651 Bq/m^2^), and the northwest slope (516 Bq/m^2^). Correspondingly, the soil erosion rate data followed the order of northwest slope (5080 t/km^2^/y) > southwest slope (4467 t/km^2^/y) > northeast slope (3973 t/km^2^/y) > southeast slope (2110 t/km^2^/y) (Figure 1B). The soil erosion rate estimated on the northwest slope was 12.06%, 21.78% and 58.47% higher than those observed on the southwest, northeast, and southeast slopes, respectively. In addition, the soil ^137^Cs inventory increased from the upper to the lower slopes (Figure 1A,B). The soil ^137^Cs content in the different positions followed the order of upper (394 Bq/m^2^) < mid (778 Bq/m^2^) < lower (917 Bq/m^2^) (Figure 1A). The soil ^137^Cs content on the lower slope was 57.07% and 15.08% higher than the contents on the upper and mid slopes, respectively. In contrast, the soil erosion rate followed the order of upper (6416 t/km^2^/y) > mid (3102 t/km^2^/y) > lower (2204 t/km^2^/y) (Figure 1B). The soil erosion rate on the upper slope was 51.65% and 65.65% higher than the rates measured on the mid and lower slopes, respectively (Figure 1B).

### 2.2. Effects of Topographic Factors on Soil Properties and Biomass

**Figure 2:**
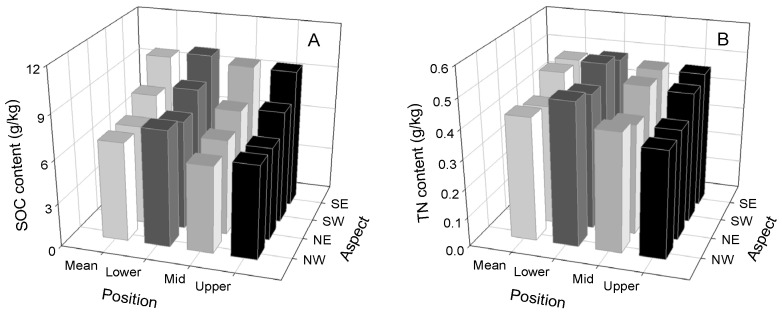
The contents of (**A**) SOC and (**B**) TN in different topographic contexts. NW: northwest; NE: northeast; SW: southwest; SE: southeast.

A descriptive statistical analysis of the soil properties is shown in Table 1. Al the soil properties except for BD varied greatly. The slope aspect factor had significant effects on SOC, clay, silt, sand, and SWC (Table 2). The lowest and highest SOC contents were observed on the northwest slope (6.81 g/kg) and southeast slope (9.76 g/kg), respectively (Figure 2A). In addition, the lowest and highest TN contents were observed on the northeast slope (0.41 g/kg) and southeast slope (0.48 g/kg), respectively (Figure 2B). Overall, the SOC and TN contents on the southern slopes (southwest slope and southeast slope) were, on average, 23.46% and 12.76% higher than those on the northern slopes (northwest slope and northeast slope). Regarding the slope factor, the SOC and TN contents increased from the upper to lower slopes by 11.83% and 15.01%, respectively (Figure 2).

The variation in soil particles in different slope aspects was consistent with the variation in soil nutrients. The slope position significantly affected the distribution of soil particles in the profile (*p* < 0.05, Table 2). The clay and silt contents on the northern slopes were 8.65% and 11.83% lower than those on the southern slopes, respectively (Table 3). Regarding the slope factor, the clay and silt contents on the upper slope were lower than those on the lower slope; nevertheless, there were no significant differences between the data from the different slope positions (*p* > 0.05, Table 2). In addition, SWC and biomass exhibited statistically significant effects on all the different slope aspects (*p* < 0.01), but we did not detect any significant differences between slope positions (*p* > 0.05, Table 2). The SWC and biomass values on the northern slopes were 28.23% and 35.88% lower than those on the southern slopes, respectively (Table 3).

### 2.3. The Relationships among Topographic Factors, Soil Erosion, and Soil Properties

**Figure 3:**
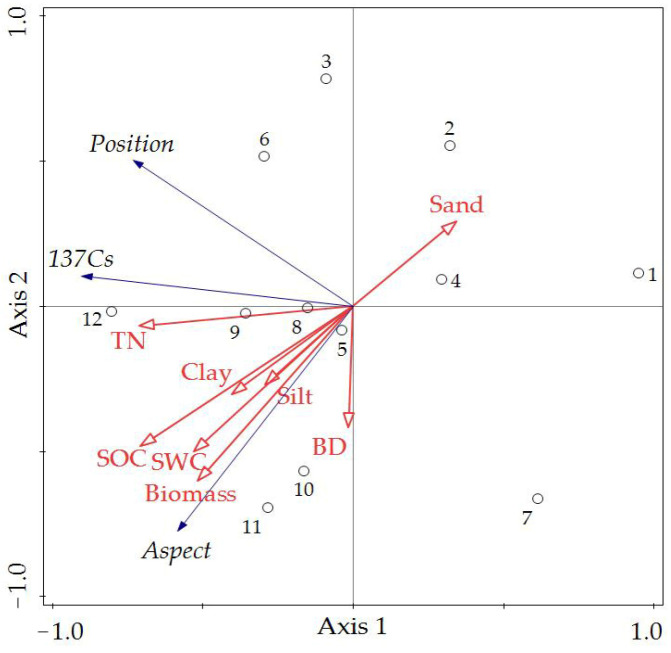
Ordination diagram of RDA on environmental factors with soil properties. The blue lines and red lines represent the environmental factors and soil properties, respectively. Numbers 1, 2 and 3 represent the northwest slope; 4, 5 and 6 represent the northeast slope; 7, 8 and 9 represent the southwest slope; 10, 11 and 12 represent the southeast slope.

We assessed the relationship between environmental factors (topographic factors, soil erosion) and soil properties using the RDA. The eigenvalues of the first and second axes accounted for 63.84% and 21.13% of the soil property variation, respectively (Figure 3). The variance in the cumulative percentage of the environmental factor–soil property relation for the first two axes was 84.97%. The correlations resulting from the RDA indicate that the variation in soil properties can be effectively explained by soil erosion and topographic factors (Figure 3). Forward selection of the variables in the RDA ordinations shows that the SOC, TN, silt, and clay contents were primarily affected by the slope aspect, but were less affected by soil erosion, while they were least affected by the slope position. Table 4 displays the relative contributions of soil erosion and environmental factors to the variation in soil properties. The slope aspect, soil erosion, and slope position accounted for 56.1%, 30.9%, and 8.4% of the variation, respectively. A Pearson correlation analysis of the correlations between the ^137^Cs inventory and the soil properties indicates a closer relationship (Table 5). There is a positive correlation between the soil ^137^Cs inventory and the measured SOC and TN contents (*p* < 0.05; Table 5).

## 3. Discussion

### 3.1. Effect of Topographic Factor on Wind and Water Erosion

According to our previous study, the wind in the study area mainly comes from the northwest, followed by the north and the west, and it rarely comes from the southeast. The annual northwestward, northward, and westward winds accounted for 91.6%, 4.8%, and 3.6% of the wind, respectively [23]. In the present study, the inequality in the wind force felt at the different slopes would lead to varying erosion rates on each of the identified slope aspects. This the main reason why the soil erosion rate on the northwest slope was 12.06%, 21.78% and 58.47% higher than that on the southwest, northeast, and southeast slopes, respectively. Moreover, slope position affects soil erosion by influencing the action of wind shear, over-land flow, and the movement of soil particles [23]. Soil particles are involved in a subprocess related to detachment, transportation, and deposition, and so as soil erosion occurs, soil losses are higher on the upper slope, but lower on the lower slope; in this context, the soil ^137^Cs was adsorbed into the fine soil particles, and simultaneously migrated [24]. The present study demonstrates that the soil ^137^Cs inventory gradually increased from the upper to the lower slopes. The minimum erosion rate was measured at the upper slope, and the maximum at the lower slope. 

### 3.2. Effects of Topographic Factor and Wind–Water Erosion on Soil Properties

Soil particles and soil nutrients are relevant indicators of soil quality, and are altered by soil erosion [25,26,27,28]. We found that the SOC, TN, and fine particle values were closely connected with the soil ^137^Cs inventory, reflecting a synergistic change trend in slope conditions. Generally, soil erosion involves a dynamic process affecting the lighter and smaller components of soil (i.e., soil fine particles), denuding and transporting the soil, resulting in surface soil coarsening and nutrient losses [6,28,29]. In the regions where both wind and water erosion occur, the higher wind erosion rate on the northwest slope meant that the contents of SOC, TN, clay, and silt were lower than those on the other slopes. Moreover, water erosion causes the migration of fine soil particles along the slopes, resulting in the contents of SOC, TN, clay, and silt being lower at the upper slopes than at the lower slopes. 

Soil moisture also affects the variation in SOC and TN contents through biotic and abiotic processes [30]. Areas with higher soil moisture contents were showed more biomass and residue, which could lead to increased nutrient inputs [30,31,32]. In addition, soil clay content is commonly associated with microbial activity, with a clear impact on SOC and TN concentrations [33,34]. Soils with higher clay contents show a smaller content of microorganisms, thus contributing to the greater resistance of the dissolved organic matter to microbial mineralization [35]. In contrast, in soils with lower clay contents, less protection is afforded to soil organic matter as a result of the higher microbial activity and mineralization [36]. The present study found soil moisture, clay content, and biomass values that were lower on the northern slope than on the southern one. As a result, the topographic factor, as a positive factor, can be said to affect soil nutrients by influencing soil erosion, the soil moisture content, clay, microbial activity, and mineralization. 

### 3.3. Implications for Vegetation Restoration

In regions where wind and water erosion occur throughout the year, soil properties that are affected by erosion processes are vulnerable, and may lead to other subsequent erosion processes occurring in sloping fields [8]. Our previous research found that wind erosion can intensify water erosion by destroying the soil structure and increasing surface roughness [9]. Wind erosion on the northwest-facing slope, in this area, was responsible for approximately 39.7% of the total soil loss, and water erosion accounted for approximately 60.3% [23]. In this context, the results indicate that wind and water could accelerate the spatial variability in erosion rate and soil properties, and cause serious reductions in nutrient content in sloping fields. Our research could serve to improve our understanding of the mechanisms of soil degradation in gully regions where wind and water erosion simultaneously occur. 

Under long-term wind and water erosion conditions, the ecological environment gradually becomes unstable and fragile. In 1999, a large-scale eco-restoration project called “Grain for Green” was launched to control the severity of soil erosion and restore degraded ecosystems on the Chinese Loess Plateau. Ecological restoration projects (including afforestation and reconverting cropland into forest and grassland) have induced a 25% increase in vegetation cover over the last decade [37,38]. Considering that topographic factors can regulate wind and water erosion rates and nutrient losses, appropriate measures should be taken, particularly focusing on northern slopes, including building bench terraces, practicing no-tillage, and enhancing vegetation coverage. It is hoped that future research could elucidate the influence of erosion caused by wind or water alone on the variation in soil properties and nutrient loss.

## 4. Materials and Methods

### 4.1. Study Area

**Figure 4:**
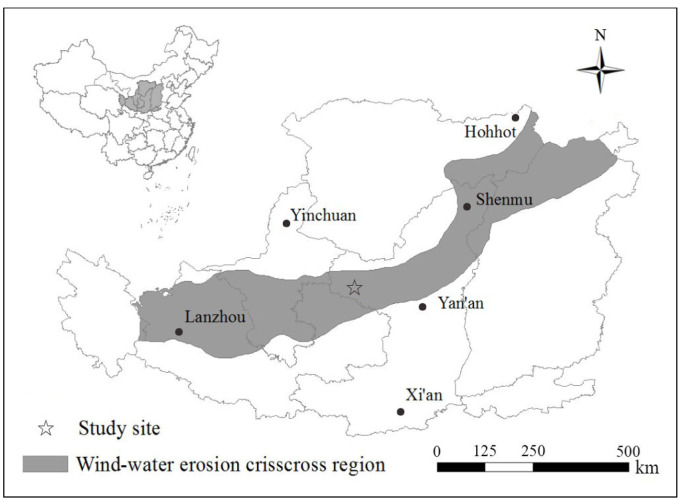
Location of the study area.

The study area was the wind–water erosion crisscross region on the Chinese Loess Plateau (103°33′–113°53′ E, 35°20′–40°10′ N; Figure 4). The climate in this region is semiarid, with an average annual temperature of 7.9 °C, an average annual rainfall of 361.9 mm, an average annual wind speed of 3.2 m/s, and an average annual frost-free period of 141 days. The average elevation of the region ranged from 1577 to 1705 m above sea level. The geomorphology of the site features undulating hills and gullies. The soil composition primarily comprises wind-deposited sediments, uniformly distributed within the profiles and ranging from 50 to 130 m deep [39]. These soils are classified as typical loessal soil and exhibit distinct features such as a lack of stratification, a silty texture, a loose structure, and microporosity. Unfortunately, the soils are highly susceptible to the significant erosion processes caused by wind and water forces. Wind erosion is particularly pronounced during March, April, May, November, and December, whereas water erosion predominantly occurs in July, August, and September.

### 4.2. Site Selection

We selected two sites with conical landforms, previously used for agriculture but later abandoned, separated from each other by less than 2 km, and the relative elevation difference between them is less than 55 m. Both study sites have more or less identical slope lengths, gradients, soil types, and land use histories (Table 6). Their absolute elevation values are both higher than those of the surrounding landforms; thus, all slope aspects (northwest, northeast, southwest, and southeast) are exposed and subjected directly to erosion by wind and water. Once agricultural processes stopped on the studied land, a local herbaceous community began to cover the soil, with *Heteropappus altaicus* Novopokr. and *Artemisia sacrorum* Ledeb. as the main herb species.

### 4.3. Soil Sampling and Analysis

We set up soil sampling plots positioned on four slope aspects (northwest, northeast, southwest, and southeast) at three slope positions (upper, mid, and lower). Twenty-four sampling plots were selected at the study sites. At each plot, first, we removed the ground litter, then took samples of the 0–20 cm-deep soil layers at three points (in an S-shaped pattern) and mixed them to make one soil sample. All soil samples were air-dried; later, they were sieved through a 2 mm screen and prepared for soil property analysis. In addition, we collected surface soils (at 20 cm) using a 5 cm-diameter hand-operated core sampler in order to determine the soil ^137^Cs contents, with three replicates performed in each plot adjacent to the original points of soil sampling. As regards measuring the other physical parameters, the soil bulk density (BD) was measured with a soil bulk sampler, featuring a 5 cm-diameter and 5 cm-high stainless-steel cutting ring, used at adjacent sampling plots. We measured the original volume of each soil core and its dry mass after oven-drying at 105 °C and surveyed the ground biomass in three evenly distributed square areas of 1 m × 1 m in each plot. In addition, as a reference, three measurements of ^137^Cs content (at 20 cm) were taken from the soil at a nearby site in an uncultivated, naturally restored *Locust* Forest. These sites had not undergone soil erosion or deposition over the last 60 years.

The determination of soil ^137^Cs was performed with a hyperpure coaxial Ge detector linked to a multichannel analyzer, detecting 662 keV peaks over a period of over 80,000 s, which yielded an analytical precision of ± 5% [40]. The soil organic carbon (SOC) content was determined via the dichromate method [41], and the soil total nitrogen (TN) content was determined with the Kjeldahl method [42]. The soil particle size distribution was determined using a Mastersizer 2000 laser diffractometer (Malvern Instruments, Malvern, England). The soil water content (SWC) was measured via gravimetry and expressed as a percentage of soil water to dry soil weight. The soil cores used for soil BD were dried to a constant mass and weighed.

### 4.4. Soil Erosion Calculation

According the data of to our previous study, the soil ^137^Cs contents of all sampling sites in this region were lower than those at the reference site [23]. Thus, the soil erosion rate can be estimated using the following equation [23,43]:

(1) $$\frac{dA(t)}{dt}=\left(1-\Gamma \right)I(t)-(\lambda +P\frac{R}{d})A(t)$$

where *R* is the erosion rate (kg/m^2^/a), *A(t)* is the cumulative ^137^Cs activity (Bq/m^2^), *d* is the cumulative mass depth representing the average plough depth (kg/m^2^), *λ* is the ^137^Cs decay constant, *I(t)* is the annual ^137^Cs deposition flux (Bq/m^2^/a), Γ is the percentage of freshly deposited ^137^Cs fallout removed by erosion before being mixed into the plow-layer, and *P* is the particle size correction factor.

### 4.5. Statistical Analysis

One-way ANOVA was used to determine the effects of the topographic factors on the soil properties and biomass, with significance determined at the 0.05 level (*p* < 0.05). The Pearson correlation coefficients were calculated to determine the correlations between the ^137^Cs content and soil properties. All the above statistical tests were conducted using SPSS version 18.0 (SPSS Inc., Chicago, IL, USA). Redundancy analysis (RDA) was applied to investigate the proportion of the variability in soil properties explained by environmental variables (Canoco 5.0, Microcomputer Power, Ithaca, NY, USA).

## 5. Conclusions

In arid and semiarid regions where wind and water erosion occur simultaneously, the soil erosion rate shows high variability in sloping fields. In this study, due to the different wind forces affecting the different slope aspects, the soil erosion rate on the northwest slope was 12.06–58.47% higher than the rates measured on the southwest, northeast, and southeast slopes. On the analyzed slopes, the soil erosion rate decreased from upper to lower levels. The variation in soil properties was closely related to soil erosion. On the northern slopes (northwest and northeast slopes), the contents of SOC, TN, clay, silt, soil water, and biomass were lower than those on the southern slopes (southeast and southwest slopes). The contents of SOC, TN, clay, and silt increased from the upper to lower slope levels. The wind–water erosion and topographic context both had significant effects on soil properties. The redundancy analysis has shown that the variation in soil properties was mainly determined by the slope aspect and to a lower degree by soil erosion. Wind–water erosion accelerates the variation in soil properties in sloping fields. These results will improve our understanding of the mechanisms of soil degradation in gully regions where wind and water erosion simultaneously occur.

## Figures and Tables

**Table 1:** Descriptive statistical analysis of the ^137^Cs inventory, soil properties, and biomass.

Variable	Max.	Min.	Mean	SD	CV (%)
^137^Cs (Bq/m^2^)	1172.59	228.26	696.22	296.18	42.54
SOC (g/kg)	10.12	6.00	7.85	1.39	17.71
TN (g/kg)	0.52	0.37	0.45	0.05	11.11
Clay (%)	11.37	8.66	9.95	0.83	8.34
Silt (%)	20.22	15.13	16.93	1.51	8.92
Sand (%)	76.21	68.42	73.12	2.26	3.09
SWC (%)	13.14	8.72	10.60	1.65	15.57
BD (g/cm^3^)	1.34	1.25	1.29	0.03	2.33
Biomass (g/m^2^)	81.00	47.22	60.49	11.65	19.26

**Table 2:** Significance levels (*p*-values are shown) of the results of the one-way ANOVA testing the effects of topographic factors on soil properties and biomass.

Topographical Factor	^137^Cs	SOC	TN	Clay	Silt	Sand	SWC	BD	Biomass
Aspect	0.020	0.002	0.196	0.002	0.003	0.003	0.004	0.512	0.013
Position	0.002	0.489	0.060	0.567	0.525	0.529	0.737	0.641	0.504

**Table 3:** Soil properties and biomass in different topographic contexts.

Aspect	Position	Clay (%)	Silt (%)	Sand (%)	SWC (%)	BD (g/cm^3^)	Biomass (g/m^2^)
Northwest	Upper Mid Lower Mean	8.66 8.94 9.31 8.97	15.13 15.83 16.56 15.84	76.21 75.24 74.13 75.19	8.98 11.14 8.85 9.66	1.32 1.26 1.26 1.28	50.95 49.10 52.73 50.93
Northeast	Upper Mid Lower Mean	9.52 10.43 10.36 10.10	15.34 15.99 17.04 16.12	75.14 73.58 72.60 73.77	9.08 8.72 8.96 8.92	1.28 1.29 1.25 1.27	49.75 47.22 57.98 51.65
Southwest	Upper Mid Lower Mean	10.91 10.67 11.37 10.98	19.06 18.07 20.22 19.12	70.03 71.26 68.42 69.90	10.67 11.14 12.16 11.32	1.31 1.28 1.32 1.30	70.42 60.20 63.58 64.73
Southeast	Upper Mid Lower Mean	9.33 9.82 10.07 9.74	16.34 16.70 16.85 16.63	74.33 73.48 73.08 73.63	11.43 12.92 13.14 12.50	1.28 1.28 1.34 1.30	62.96 79.99 81.00 74.65

**Table 4:** Statistical summary and canonical coefficients of environment factors affecting soil properties’ variations for the first two axes of the redundancy analysis.

Environment Factor	Axis 1	Axis 2	Lambda−1	Lambda−A	*p* Value
Aspect ^137^Cs inventory Position	0.9618 * 0.7533 * 0.2639	−0.2456 0.2846 0.9492 *	56.1 30.9 8.4	56.1 8.4 2.5	0.002 0.016 0.508

* indicate significance at the 0.05 and 0.01 levels, respectively. Lambda−1: the variance when the variable is used as the only factor. Lambda−A: the additional variance each variable explains when it is included in the model. *p* value is the significance of Lambda−A.

**Table 5:** Correlation analysis of soil ^137^Cs inventory and soil properties.

	^137^Cs	SOC	TN	Clay	Silt	Sand	SWC	BD	Biomass
^137^Cs SOC TN Clay Silt Sand SWC BD Biomass	1 0.658 * 0.619 * 0.324 0.205 −0.256 0.479 0.045 0.506	1 0.782 ** 0.258 0.368 −0.341 0.740 ** 0.333 0.863 **	1 0.480 0.699 * −0.643 * 0.593 * 0.076 0.635 *	1 0.862 ** −0.941 ** 0.260 0.250 0.358	1 −0.983 ** 0.426 0.286 0.441	1 −0.378 −0.282 −0.425	1 0.418 0.820 **	1 0.441	1

* and ** indicate significance at the 0.05 and 0.01 levels, respectively.

**Table 6:** Detailed information of the study sites.

Site	Slope Aspect	Slope Gradient (°)	Slope Length (m)	Altitude (m)
1	Northwest Northeast Southwest Southeast	17 19 18 20	70 70 70 70	1645 1645 1645 1645
2	Northwest Northeast Southwest Southeast	18 17 19 21	80 80 80 80	1590 1590 1590 1590

## Data Availability

All data are presented in the main text.
